# Social Network Analysis Applied to a Historical Ethnographic Study Surrounding Home Birth

**DOI:** 10.3390/ijerph15050837

**Published:** 2018-04-24

**Authors:** Elena Andina-Diaz, Mª Antonia Ovalle-Perandones, Ignacio Ramos-Vidal, Francisca Camacho-Morell, Jose Siles-Gonzalez, Pilar Marques-Sanchez

**Affiliations:** 1 Health Research Group, Welfare and Social and Health Sustainability (SALBIS), Faculty of Health Science, University of León, Vegazana Campus, s/n, 24071 León, Spain; 2 Library and Information Science Department, Faculty of Humanities, Communication and Documentation, Carlos III University, 28903 Getafe, Madrid, Spain; movalle@bib.uc3m.es; 3 Social Psychology Department, University of Seville, 41004 Seville, Spain; iramos5@us.es; 4 School of Social and Human Sciences, Pontifical Bolivarian University, Medellín, Colombia; 5 Delivery Room, La Ribera University Hospital, 46600 Alcira, Valencia, Spain; francisca.camacho@uv.es; 6 Faculty of Nursing and Podiatry University of Valencia, 46010 Valencia, Spain; 7 Faculty of Health Sciences, University of Alicante, 03690 San Vicente del Raspeig, Alicante, Spain; jose.siles@ua.es; 8 Health Research Group, Welfare and Social and Health Sustainability (SALBIS), Faculty of Health Science, University of León, Ponferrada Campus, s/n, 24401 Ponferrada, León, Spain; pilar.marques@unileon.es

**Keywords:** social network analysis, home birth, midwife, ethnography, history

## Abstract

Safety during birth has improved since hospital delivery became standard practice, but the process has also become increasingly medicalised. Hence, recent years have witnessed a growing interest in home births due to the advantages it offers to mothers and their newborn infants. The aims of the present study were to confirm the transition from a home birth model of care to a scenario in which deliveries began to occur almost exclusively in a hospital setting; to define the social networks surrounding home births; and to determine whether geography exerted any influence on the social networks surrounding home births. Adopting a qualitative approach, we recruited 19 women who had given birth at home in the mid 20th century in a rural area in Spain. We employed a social network analysis method. Our results revealed three essential aspects that remain relevant today: the importance of health professionals in home delivery care, the importance of the mother’s primary network, and the influence of the geographical location of the actors involved in childbirth. All of these factors must be taken into consideration when developing strategies for maternal health.

## 1. Introduction

Ever since the dawn of humanity, birth has been considered a natural, biological and female act but also a social and cultural one. Mothers gave birth in their homes, surrounded by members of their primary network and usually attended by other women known as traditional midwives or traditional birth attendants (TBAs). In most parts of the world, TBAs generally performed similar roles, functions and actions. They did not usually have official health care training, but did have extensive skill and experience, and based their interventions on local cultural beliefs and practices. This situation slowly changed over the centurie as the work carried out by these women was gradually professionalised, giving way to qualified midwives.

In addition, male physicians began to attend births in cases of extreme gravity. In the 20th century, major social and health changes occurred throughout the world, transforming the way that health was perceived and treated. Thus, delivery went from being attended exclusively by TBAs to being the preserve of trained health professionals (midwives and doctors). As the scenario changed from the mother’s home to the hospital, the members of her primary network (family and friends) who had formerly accompanied her could no longer do so because their access to hospital delivery rooms was restricted.

Delivery care in developed countries began to change substantially in the mid 20th century, and Spain was no exception. Previously, health professionals provided care for the poor through charities, while the rest of the population, the majority, paid for their services. One of their functions was attend home births. Providing this care in rural areas was problematic due to the travelling involved, the unhygienic conditions encountered and the scant economic remuneration health professionals received. Consequently, deliveries were attended by TBAs (in Spain they were called “parteras”) or by members of the mother’s primary network, as had happened in the past. However, in 1942, a new level of health care was instituted known as compulsory sickness insurance (Spanish initials: SOE), for insured workers and their families. One of the benefits was home birth care. In the 1950s and 1960s, the number of people entitled to SOE services rose, and more and more women were attended at home by health professionals, as well as by TBAs. At the same time, large hospital infrastructures began to be built, and women gradually started going to these to give birth, until by the 1970s, virtually all women covered by SOE gave birth in hospitals [1].

The generalisation of hospital births in Spain and throughout the world improved safety during birth, yielding substantially better perinatal outcomes. In parallel, however, delivery became increasingly medicalised. This led to unnecessary levels of intervention in most births (it is estimated that only 10% of all births require induction), a practice which soon received severe criticism. Thus, between 1980 and 1990, the World Health Organisation (WHO) published a series of guidelines on delivery care, recommending as little medical intervention possible and use of the appropriate technology for each type of delivery [2,3]. Rather than technology, women in labour needed a comfortable and welcoming physical environment where their freedom and privacy was respected, and discrete emotional support that made them feel safe without being watched. Universal hospital delivery was one of the practices that the WHO recommended abandoning.

As a result of these recommendations, recent years have witnessed a growing interest in home births [4,5,6,7]. In many European countries, however, the percentage of home births remains low, accounting for between 1 and 10% of deliveries, with the exception of the Netherlands, where they account for up to 30% of deliveries [8].

One of the advantages of a home birth is that it enables the mother to receive continuous support throughout labour. This is provided both by health professionals, basically a midwife, and by members of the mother’s primary network (family and friends), since these can be present. Continuous support yields benefits for both the mother and her newborn infant, and is therefore also being encouraged in hospitals [9,10,11,12].

Another advantage of a home birth is respect for women’s physical, emotional and cultural dimensions [13]. As the WHO has argued, home births offer women a comfortable and welcoming physical environment where their freedom and privacy is respected. There is strong evidence in favour of a planned home delivery for women with low risk pregnancies [14].

Given the current level of interest in home births, it might be pertinent to perform a retrospective study that sheds light on the mother-TBA-health professional relationship in the past. An analysis of the relational structures that arose around delivery could yield answers to the following questions: why did pregnant women decide on one or another form of delivery care? And how did environmental characteristics favour or hinder changes in the place selected for delivery? These questions implicitly concern several aspects that have received little attention in the literature, namely the contacts and relationships between individuals which could influence given behaviours and decisions. In other words, besides individual factors, it is also necessary to consider the social systems and norms that are created through connections or links between people [15].

Social network theory can help elucidate these structural systems, which are formed by people (actors) and relationships (ties) and give rise to networks through which resources are transferred. Some of the core ideas of this theory are that: (i) people think, feel and act according to the pattern of ties between actors, (ii) behaviours must be analysed in light of the ties between actors during the period studied, (iii) the unit of analysis is the individual and his or her ties [16]. These aspects can be studied via the method known as social network analysis (SNA), which is based on graph theory and disciplines such as anthropology, psychology and sociology. For example, SNA examines the type of tie, its direction and intensity, the importance of position and the subgroups that can form in social networks [17].

There are several studies in literature which have applied SNA to the community context, the subject of the present research. Luke and Harris [18] have expressly encouraged researchers to apply SNA to topics such as the advancement of health through communities based on coalitions, or the support provided through structures formed by family, neighbours and friends in the community. SNA has also been applied to health promotion and prevention campaigns, citizen empowerment strategies and recruitment of key actors with the capacity to influence community health [19].

Regarding SNA studies applied to maternity care, the subject of the present research, the literature provides little evidence [20,21,22]. To date, it has been found that community-based midwives present a high degree of connectivity in the community, suggesting that they occupy a position in the network that facilitates ties between different professionals, thus endowing them with a standing that renders them competent to act as maternity case managers [20]. However, no in-depth analyses have been conducted of the relational processes that characterise actors with a higher standing and thus this relationship strategy has yet to be determined, rendering it impossible at present to promote or replicate it with other actors in similar contexts. There is also a lack of historical studies analysing the inter-professional relational processes that have occurred in various societies, or interpreting possible collaborative tensions that have influenced current practice [23]. A rapid retrospective glance suggests that almost all research attention has been focused on the influence of the most direct contacts, and little on the influence of indirect contacts that may arise from direct interpersonal relationships [24].

Another question associated with SNA and of interest in the present research concerns the connections between actors and geographical or even urban aspects of the community, since these could explain processes of trust and cohesion. Do subgroups or small communities form within the local community? What interests do they represent? What characteristics are presented by the places where deliveries are usually attended by TBAs or the mother’s primary network? In this respect, it has been found that the log-odds of friendship existence decrease with the logarithm of distance [25]. This suggests a relationship between social support, distance to travel within the community and social structures. Elucidating this idea concerning social support, distance and geographical location, etc., would contribute to planning network interventions to improve maternity care. Community networks have proved useful for conceptualising self-care in everyday contexts [26]. They provide structures through which to transfer help, advice, information and even emotional resources that are very beneficial to pregnant women in their social environment.

Hence, knowledge obtained from the perspective of social networks concerning the human interactions and social interdependencies that arise between professional, traditional actors (TBAs) and mothers during delivery could indicate areas for improvement that would have a direct or indirect impact on the physical, psychological and social care of pregnant women. Such a perspective could also contribute to planning maternal health strategies, seeking external factors that could replace current delivery practices with other, more appropriate ones [22].

To shed light on the relational phenomena that arose around delivery, we conducted a descriptive study of a historic event: the transition from home to hospital births that occurred between the 1940s and 1970s in Spain. Many other parts of the world also experienced this shift from home births attended by TBAs and/or health professionals, to hospital deliveries. We reconstructed this relational past focusing on a rural area in Spain and using oral sources (the personal experiences of women who had given birth in that period and geographical location).

Thus, our objectives were:

- To confirm the transition from a home birth model of care to a scenario in which deliveries began to occur almost exclusively in a hospital setting.
- To define the social networks surrounding home births.
- To determine whether geography exerted any influence on the social networks surrounding home births.

We decided to use structural evaluation techniques because the SNA is a theoretical and methodological paradigm that allows us to (a) evaluate the relational context in an empirical way; and (b) capture contexts of social interaction, which (c) determine the behavior of the actors that are part of that context [18]. We used SNA in order to facilitate the visualization of the structural contents.

## 2. Materials and Methods

The theoretical framework, selection of the group, access to the scenario and data collection all reflected a qualitative approach (historical ethnography).

### 2.1. Location

The study was carried out in the municipalities of Almanza and Cebanico (León province, NW Spain). These cover 231.74 km^2^ and include 16 villages. In 1940, they had 3944 inhabitants [27], who were mainly employed in agriculture and livestock farming.

### 2.2. Description of the Sample

The sample consisted of 19 women who had given birth at home in the 1940s–1970s, in the study area. We conducted purposive sampling based on criteria such as accessibility, interest in the research, ease and feasibility. We employed the snowball method to contact people who knew the area and circumstances and could suggest others. To ensure quality and rigour, we included participants until reaching saturation [28].

### 2.3. Data Collection

Data were mainly collected in semi-structured interviews for which an ad hoc interview guide was drawn up. Since this study formed part of a larger project, we selected the responses that were related to the study objectives.

The interviews were carried out in the women’s homes once they had signed an informed consent form, and were attended by a member of the research team, the woman interviewed, and sometimes, one of her relatives. To facilitate subsequent transcription of the interviews, these were tape-recorded.

The women were interviewed mainly between April and September 2012. The interviews took place in their homes.

Several interviews were conducted with each participant, until reaching saturation.

The researcher responsible for collecting data in the field was a long-term resident in the area, whose occupation and personal circumstances facilitated contact and interaction with participants to obtain information.

To accurately identify the network of health professionals working in the area, we consulted various sources in the Provincial Archives of León and the archives of the Nursing College of León (León, Spain).

### 2.4. Variables

We employed a social network analysis method. A network basically consists of a graph, its ties and additional information from the vertices [29]. Among many other ways to consider them, networks can be classified on the basis of their mode. In this context, mode refers to the number of groups of elements used to measure structural variables. In line with this criterion, there are one-mode networks and two-mode networks. The former are the most common and only represent one set of actors, whereas two-mode networks represent two sets of actors, or a set of actors and a set of events, and are called then affiliation networks.

In the present study, the childbirth care networks identifying ties between the interviewees and the people who attended them during childbirth are two-mode networks. These were analysed using two different methods [30]. On the one hand, home birth care networks were studied using the direct method, characterised by considering both modes as a single set. This prevented loss of information. On the other hand, geographical influence networks were analysed using the conversion method, where the original two-mode networks were converted to one-mode networks. This method focuses on the ties that form part of one of the modes.

This dual perspective enabled us to use one- and two-mode metrics. In addition, these metrics were used at two levels of analysis: a macro-level (the complete network) and a micro-level (nodes and subgraphs).

- Micro-level
  - Degree: this measure considers node centrality, quantifying how many ties it has. The value for interviewee degree quantifies the number of ties with two-mode nodes or instances of care received. The degree of two-mode nodes or carers reflects the instances of care these people provided. Although this can be normalised, here we give absolute values.
- Macro-level
  - Number of nodes: this measure quantifies the size of a network, which in the case of two-mode networks is considered to be the number of nodes included within each mode: in this case, the interviewees (mode one) and the birth attendants (mode two).
  - Completeness: this measure quantifies the number of possible ties, and in a two-mode network in which ties between nodes in the same mode are not allowed or are occasional, it quantifies the maximum number of ties that are defined by the product of the number of one-mode nodes and the set of two-mode nodes, divided by 2 when the ties between nodes are reciprocal (not directed).
  - The number of existing ties: this measure refers to the number of ties between nodes in both modes. The value is obtained by counting the total number of ties that occur in the network analysed [31].
  - Graph components: these are parts that are internally connected but at the same time, disconnected or isolated from other parts, and must thus be considered subgroups, substructures or subgraphs of a network [32].
  - Network cohesion: this is measured from two perspectives: average degree and density. Both indicators are suitable to measure cohesion and both allow a comparison between networks, without being affected by the size or number of nodes in each one of them [33]. The average degree is the arithmetic mean of the absolute degrees of the nodes [34]. Density reflects the proportion of possible ties relative to the ties that actually appear in the graph [31] and is also the arithmetic mean of the normalised average degree of the nodes [35]. Density values for a network can range from 0 (graph without ties) and 1 (complete graph).

### 2.5. Data Analysis

We carried out a content analysis with the collected data. From this analysis, we deduced relational data. These relational data were the base for constructing the network (retrospective construction). Matrices were created from the information obtained, including interviewees in the first mode and those who attended home births (health professionals, TBAs and family/friends (relatives)) in the second mode. To create a matrix of geographical influence, we used information on the municipality in which the interviewees had resided and given birth. The resulting matrices were analysed using Pajek (version 5.03) and UCINET (version 6.623) software for social network analysis. For graphs with networks formed by more than one component, we used the Kamada-Kawai algorithm (separate components). For the other networks, we used the original circular algorithm. Both are appropriate and implemented on the software used.

In addition, we consulted the answers obtained in the interviews, as well as the information obtained from the archives, to confirm the data obtained from the SNA.

### 2.6. Ethical Considerations

An informed consent form was given to all the women interviewed. This explained the voluntary nature of participation, and guaranteed the confidentiality of the data obtained. Personal names were removed from the data obtained from archival sources, as well as place names that could identify the subjects (Law 6/1991, 19 April, on archives and documentary heritage of Castile and León, Spain).

National and international guidelines (Code of Ethics and Declaration of Helsinki) were adhered to at all times. We observed the legal provisions regarding data confidentiality (Law 15/1999, 13 December, on the protection of personal data).

This study was approved by the Ethics Committee of the University of Alicante (Spain) (UA-2015-08-18).

## 3. Results

### 3.1. Trends over Time in Home and Hospital Births

**Figure 1:**
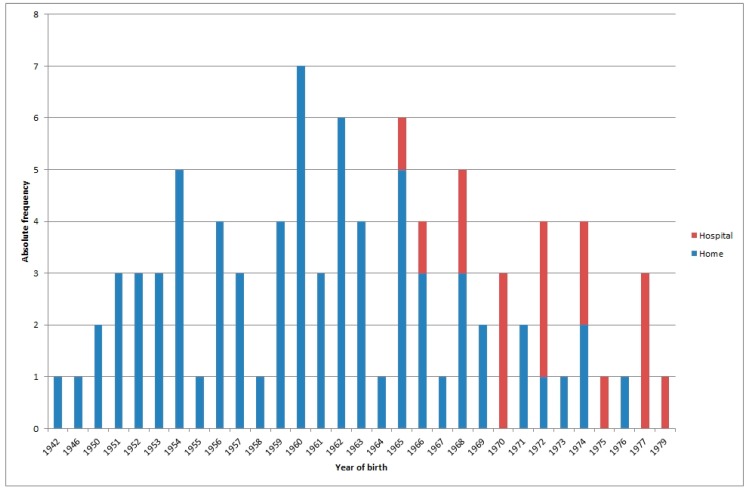
Trends over time in home and hospital births: 1942–1979.

Figure 1 shows the places where the 19 interviewed women gave birth between the 1940s and 1970s. Three different periods can be distinguished: from 1942 to 1964, when deliveries occurred at home; from 1965 to 1977, when births occurred at home or in hospital, with no definite pattern; and from 1977 onwards, when deliveries occurred in hospital.

Using the direct method (which considers ties between nodes of the modes without any loss of information), we analysed the care networks that arose in the interviewees’ homes when they gave birth.

### 3.2. Care Network When Delivery Occurred at Home

**Figure 2:**
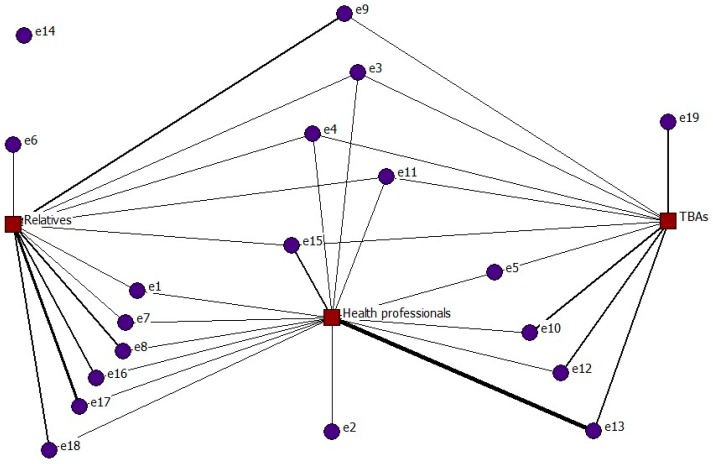
Two-mode network of home birth care provided by health professionals, TBAs or relatives.

Figure 2 presents a two-mode network formed by a total of 22 nodes, of whom the 19 shown in purple correspond to the interviewees (first mode) and the 3 nodes shown in red are the participants involved in care (second mode): health professionals, TBAs and family/friends (relatives).

The interviewees’ ties with the second mode presented a frequency equal to one in 16 cases, and greater than one in 21 cases (darker grey lines). As an example of this group, a tie greater than one indicates that the same woman was attended by TBAs during several of her deliveries.

Whenever the women were attended in their homes by a doctor, nurse (in Spain, in this time, they were called “practicante”) or midwife, this is reflected in the network by a tie with the health professionals node (relations 14). For deliveries attended by a person versed in local practices and knowledge concerning delivery, this is indicated by a tie with the TBAs node (relations 10). Births supported by relatives, neighbours or friends are shown by a tie with the relatives node (relations 12).

Node e14, which is isolated, corresponds to one interviewee who received no support during her 17 deliveries (self-care).

To conclude with this network, note that 15 of the 19 women contacted more than one actor to attend their deliveries. This can be interpreted as indicating that they knew about the alternatives for giving birth, and chose according to circumstances.

The next step was to decompose the network into the networks resulting from the tie between the first mode and the three groups in the second mode.

For each level of analysis, the health professionals networks were analysed according to their occupational category. When the objective of the networks was to analyse the geographical influence of health professionals, these are shown in the network anonymously and individually. In the TBAs network, these also appear anonymously and individually. In the relatives network, these are grouped according to their tie or relationship with the interviewees. 

**Figure 3:**
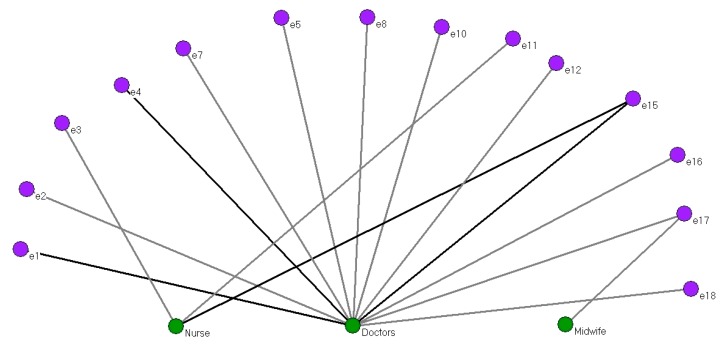
Two-mode network of care provided by health professionals (doctor, nurse or midwife).

**Figure 4:**
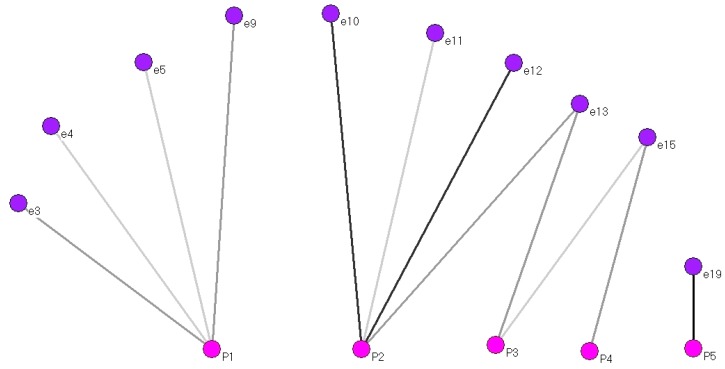
Two-mode network of care provided by TBAs when delivery occurred at home.

**Figure 5:**
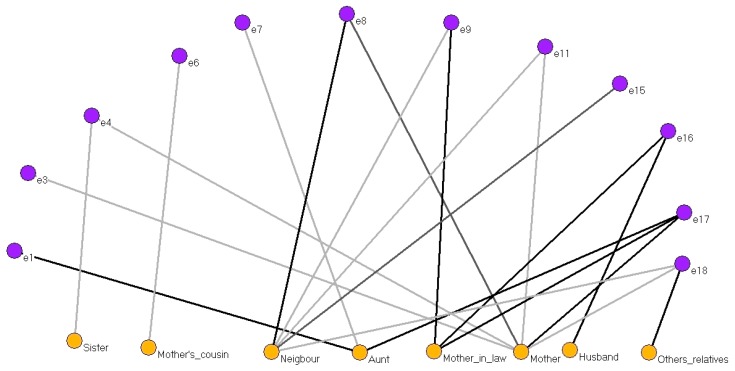
Two-mode network of care provided by family members or friends when delivery occurred at home.

[The values of the metrics for the three networks (Figure 3, Figure 4 and Figure 5) after decomposition are available on https://tinyurl.com/ijerph-sna-surrounding-table1].

For mode 1, the number of nodes metric indicates the interviewees who were attended by each of the three types of actor. The actors who attended most interviewees were health professionals (14 interviewees), followed by relatives (12 interviewees) and lastly the TBAs (10 interviewees). The number of actors in these three networks represents the degree of the two-mode nodes in the two-mode network of care provided by health professionals, TBAs or relatives when the interviewees’ deliveries occurred at home. The same metric for mode 2 indicates the possible care choices or opportunities. In other words, the interviewees were attended by health professionals belonging to three different categories (doctors, nurses or midwives); by five different TBAs (anonymous and individual); or by eight different categories of relatives (mother, aunt, mother-in-law, husband, etc.). Given the limitation explained, this measure should not be used to compare the three networks.

The metrics of completeness and number of existing ties can be used to assess the possible and actual ties between the nodes in both modes. Again, the three networks should not be compared, but considered individually. In the health professionals network, both metrics are proximate due to the grouping of the nodes into three professional categories. In this case, the node with the highest number of relations must be considered, which corresponds to doctors. In the case of the TBAs network, the relationship between both values is reduced by almost half. This is explained by the existence of 3 components in the network, which implies that the TBAs were acting in a more individual manner, with no connection between some of them. Lastly, in the relatives network, the difference between completeness and number of existing ties is due to the number of nodes in the second mode, which increases the number of possible ties.

For the number of existing ties (ties = 1 or >1) in these networks, 1 indicates an occasional tie and greater than 1 a repeated tie. In the health professional network, the number of ties = 1 is considerably higher than >1. This indicates that the interviewees occasionally asked health professionals to attend their deliveries (according to the interviewees’ answers, this occurred when deliveries presented a complication requiring medical intervention). The TBAs network presents a higher number of ties >1, which we interpret as indicating satisfaction with their work (as confirmed by the answers, interviewees called TBAs due to affection, proximity and recognition). The relatives network presented a similar number of values = 1 and >1, possibly indicating a diversity of choices and varying circumstances. For example, if a family member or friend (e.g., the mother) was absent during one delivery, another might attend (e.g., the mother-in-law), but if the first was once again available on another occasion, he or she would attend.

As regards network cohesion, measured both by density and average degree, the health professional network was the most cohesive. Although the relatives network presented a higher average degree, it obtained a lower value for density.

Among the two-mode nodes, doctors obtained the highest degree centrality in the health professionals network, mothers in the relatives network, while in the TBAs network, TBAs 1 and 2 obtained the same value.

### 3.3. Health Professional Care Network (Doctor, Nurse or Midwife) When Delivery Occurred at Home

Figure 3 shows a two-mode network formed by the 14 interviewees (purple) who received care from a health care professional (two-mode nodes, indicated in green: doctor, nurse or midwife).

Most of the women interacted with the two-mode nodes on a single occasion (12 lines). Four interviewees did so on more than one occasion (dark grey lines). Such was the case of e1 and e4 with the doctor, for example. Complementing the data with the answers given by the women’s interviewed, these instances corresponded to deliveries that presented a complication requiring medical intervention.

In our entire study, only one interviewee on one occasion was attended by a midwife as well as by a doctor (e17), while three other women were attended by a nurse (e3, e11 and e15, this latter simultaneously with a doctor).

Thus, doctors were the health professionals who offered most support at home births, attending 12 interviewees. The more important role of the doctor in contrast to the other health professionals is in line with the recognised role of each of these categories.

It should be noted that five of the interviewees had never had access to care from health care professionals or had deliveries without complications and thus did not require care.

### 3.4. TBA Care Network When Delivery Occurred at Home

Figure 4 shows the 10 interviewees (purple) who received care from TBAs (two-mode nodes, pink) during a home delivery.

The network consists of three components. The component that includes TBA P1 is on the left. The main or largest component in which the interviewees interacted with three TBAs (P2, P3, P4) is in the centre. An isolated component where interviewee e19 was attended by TBA P5 is on the right.

The highest degree centrality corresponds to TBAs P1 and P2, and the case of TBA P2 is notable due to her frequency, as she attended interviewees e10 and e12 during four of their deliveries.

The structure of this network indicates that sharing experiences or information about delivery (by the TBAs or the interviewees) was only possible between the nodes of the main component (P2, P3, P4). The possibilities of sharing experiences in the nodes where P1 or P5 appear were minimal.

### 3.5. Relative Care Network When Delivery Occurred at Home

Figure 5 shows the interviewees (12 nodes, purple) who received care from a family member or friend (8 two-mode nodes, yellow) during delivery.

With the exception of husbands and family members or friends whose sex was not specified, all the two-mode nodes were female, explained (according to the interviewees’ answers) by the social and cultural circumstances of the time.

Although the interviewees referred to these people as close family members or friends rather than TBAs, in fact they were performing a task similar to that of the TBAs. Interviewees identified them according to their relationship with them: cousin, sister, mother-in-law, aunt, neighbour or mother.

### 3.6. Geographical Influence Network

In reporting the results, we will continue to use the previously mentioned method of conversion to describe the interest underlying the ties that form part of the different modes. Consequently, these are addressed as one-mode networks.

#### 3.6.1. Network of the Geographical Influence of TBAs Due to Co-Care

Below, we analyse the influence of TBAs in the localities where the interviewees gave birth, in other words, co-care in deliveries.

**Figure 6:**
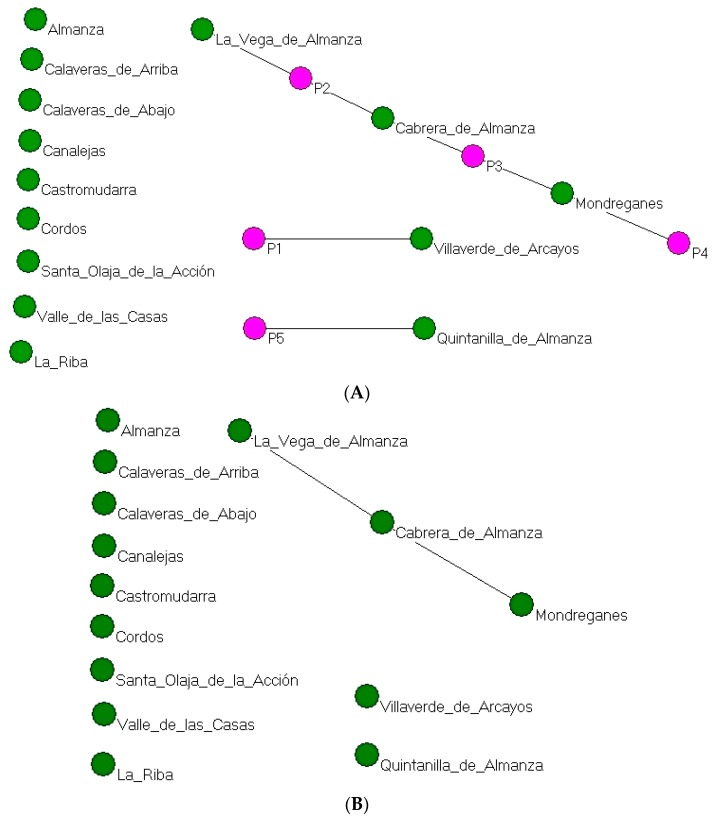
(**A**) Two-mode network of municipalities in which deliveries occurred and the TBAs who attended them (pink: TBAs; green: localities); (**B**) One-mode network of municipalities by co-occurrence of deliveries attended by TBAs.

Using a matrix with two modes, TBAs and localities, a value was assigned to each cell according to the number of births attended by each TBA in each municipality. This is consequently a weighted, 5 by 14, two-mode matrix. The conversion process loses information from the general context in which ties occurred but can be used to identify co-occurrence (in this case of TBAs who attended births in the same municipalities) and thus obtain a network of ties between municipalities due to co-attendance or co-care on the part of TBAs. The network in Figure 6A shows that TBAs P2 and P3 were the most influential, simultaneously covering two localities that were only a few kilometres apart. Hence, in this case, being more influential was associated with a better geographical location. In addition, the villages of La Vega de Almanza, Cabrera de Almanza and Mondreganes were connected because they shared TBAs.

In the one-mode network of municipalities linked by the co-occurrence of deliveries attended by the TBAs in Figure 6B, the number of isolated municipalities increased. To the municipalities that in the previous two-mode network were already isolated must now be added those which did not share a TBA. The resulting network has a density of 0.39 and due to its characteristics, no grouping procedures or others with a similar purpose were applied.

#### 3.6.2. Network of the Geographical Influence of Health Professionals Due to Co-Care

As with the analysis of geographical influence due to TBA co-care, we will now analyse the influence of health professionals in the interviewees’ area. Each of the births occurred in a given municipality, and when a health professional attended, a tie between localities was established due to co-care in deliveries attended jointly by a doctor (DR), nurse (PA) or midwife (MID).

**Figure 7:**
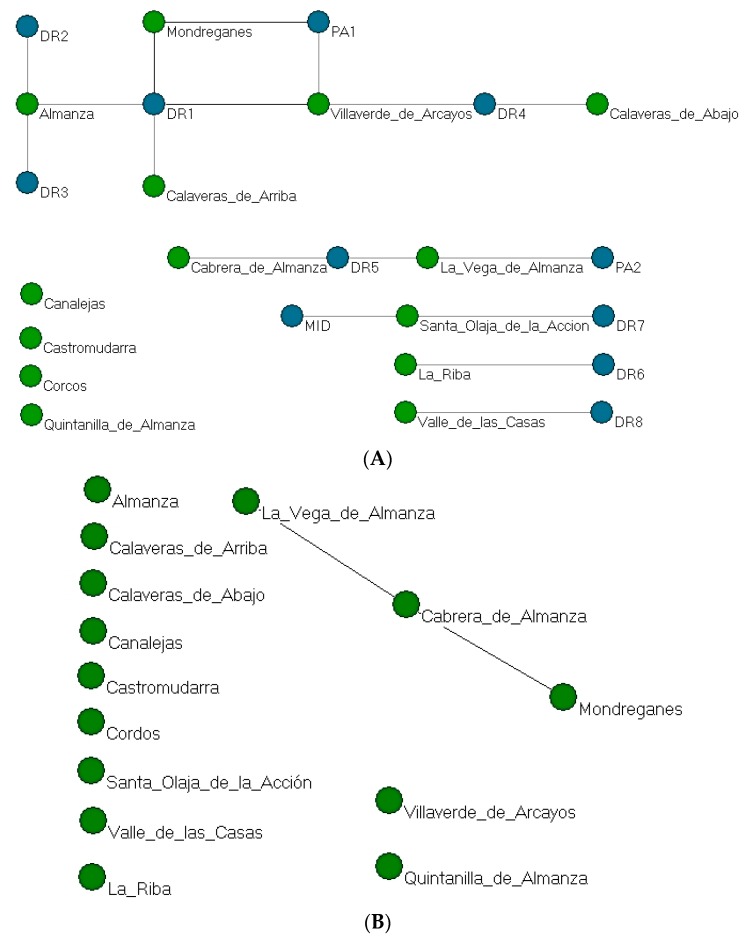
(**A**) Two-mode network of municipalities in which deliveries occurred and the health professionals who attended them (blue: health professionals; green: localities); (**B**) One-mode network of municipalities according to co-occurrence of deliveries attended by health professionals.

The two-mode network in Figure 7A shows the co-occurrence in this case of health professionals (11) who attended deliveries in the different municipalities (14), yielding a network of ties between municipalities due to co-attendance or co-care on the part of health professionals. When interviewees named a health care professional generically, the information was also considered in this way.

This graph confirms that there were various ties between the localities, primarily due to the mobility and attendance of doctor DR1 in different villages. To a lesser extent, nurse PA1 also played a role in linking the villages, influencing flows between Mondreganes and Villaverde de los Arcayos.

Having dichotomised the two-mode matrix, the one-mode network in Figure 7B presents a density of 0.14 and there are a total of 26 ties between different villages as a result of health professionals’ actions.

Due to the characteristics of the network, we conducted a cluster analysis following the optimisation procedure [36,37], which yielded a total five clusters, mostly formed by two municipalities. We obtained a good fit for the final solution [(n = 5 clusters); R^2^ = .517, fit index = .281]. Although we have mentioned five clusters, three of these consist of municipalities that appear in isolation in the graphs, and thus it is the remaining two clusters that are of relevance. One includes Vega de Almanza and Cabrera de Almanza, and the other Almanza, Villaverde de Arcayos, Calaveras de Abajo, Calaveras de Arriba and Mondreganes.

A comparison of the two geographical influence networks (TBAs and health professionals) reveals that health professionals (specifically, doctors) generated the most connectivity, whether between women (two-mode networks) or between localities (one-mode geographical influence networks). In addition, the emergence of clusters in the health professional network but not in the TBA network further supports this idea of connectivity. This is repeated at all levels of analysis and contrasts with the perception expressed by the interviewees in their interviews, in which they stated that their relatives network was the most significant one during delivery.

## 4. Discussion

Here we will discuss several ideas or aspects that have emerged from our results.

The first of these is that the transition from the home birth model of care to the hospital delivery model occurred slowly and gradually between the 1940s and 1970s, as it did in other parts of the world [38]. Widespread adoption of the SOE in Spain and the construction of large hospital infrastructures prompted a decisive shift to hospital delivery in the 1970s [1]. This paradigm change occurred later in rural areas due to their specific economic, social and geographical circumstances [39]. In our study area, home births persisted until the late 1970s.

The second idea concerns the care networks that formed around home births. Of these, the health professional network was the most cohesive, presenting a greater probability of connecting actors. This emerged from an analysis of the SNA metrics (degree, density, average degree and components) for the home birth care network. Interviewees had more opportunities to interact, and actually did interact more, with health professionals than with the other actors (TBAs or relatives). In fact, doctors were the actors who attended most interviewees, followed closely by family/friends, and lastly by TBAs.

These findings may seem surprising since in their interviews, the interviewees [40] said that they felt closest to people in the domestic sphere (TBAs and relatives) due to cultural and emotional affinities. These were also the first people they called to attend their deliveries, whereas health professionals were called in the case of complications.

Although our results should be interpreted with caution due to the different way of addressing the health professional, TBA and relatives networks, they invite reflection on the interviewees’ discourse regarding birth as a natural physiological process. Focusing on a time of social change when birth ceased to be a physiological event and became a medical event, SNA has enabled us to contrast the perceptions of these women and their primary network (about the physiological nature of birth) with their behaviour or actions, aimed at seeking professional health care.

Furthermore, SNA has highlighted the role of doctors within the home birth care network. This finding should not be overlooked today, when various organisations and associations are suggesting a return to the home as the most appropriate setting for delivery [4,5,6,7,8,14]. In many countries, health care offers several delivery options: hospital units, birth centres or at home [8,41,42]. This phenomenon is explained by the privacy, dignity, freedom, comfort and respect for the mother’s emotional and cultural dimensions [2,3,13]. However, in the 21st century, it should not be forgotten that home births must be attended by trained professionals to ensure a safe delivery. It is no longer doctors, but qualified midwives specifically trained in home births, who should attend home deliveries.

The third idea is related to the network of family members or friends who helped the interviewees give birth. This network contained a wide diversity of actors. Regardless of their relationship to the interviewee (e.g., mothers, husbands, sisters), these people played a key role during delivery, and one very similar to that of the health professionals. In this regard, many recent studies have indicated the importance of the mother’s primary network in providing companionship and emotional and cultural support [9,10,11,12]. In our study, most of these family members and friends were women. Although the importance of the female support network in our study can be explained by the social and cultural circumstances of the time, other studies have also found that pregnant women are most frequently accompanied by women during delivery [10,43].

The fourth idea concerns the possible influence of geography on home birth networks. In the geographical influence networks of TBAs and health professionals, we found that both (but above all the latter) generated ties between localities and between women through the care that they provided simultaneously in several places. This function of connectivity was decisive in this rural area where communication between localities was difficult. In terms of the present, it might be useful to consider this function of connectivity when planning optimal geographic locations for a health service or a health professional, to promote equitable access [44,45,46]. This is especially important in rural areas that are difficult to access. In this respect, Vieira et al. have indicated that use of health services during delivery is determined by distance or transport [47].

Another cross-cutting aspect examined in the present study was the community dimension of home births. Although care was often provided in the mother’s home, the health professionals’ interventions generated indirect medical care ties between localities. This finding seems to suggest that it was not the resources of the TBAs or families that were deployed to mitigate any risks entailed in delivery, but rather a social and community network of support and resources in which various people in the community necessarily intervened (e.g., a driver or car owner to drive the doctor from one village to another; a chemist to provide the family with medical supplies and someone responsible for notifying a doctor by telephone or radio in the case of an emergency). In short, the provision of care in rural villages by health professionals and TBAs generated a sort of social capital in the community, eventually forging a network of formal and informal contacts that was activated to meet social and health needs. This finding demonstrates the importance of informal support resources in the provision and quality of health care and social services [48].

Along the same lines, we obtained another interesting result from the SNA. A comparison of the home birth care network of TBAs with that of health professionals revealed that the TBAs were acting in isolation, as their network was composed of 3 components. This hindered the dissemination of information and innovation. However, health professionals (whose network was composed of a single component) presented greater connectivity, a greater exchange of information and therefore, more resources and innovations. In terms of the present, this finding suggests the need to think about and analyse how the structure of relatives and/or health professional networks may promote the exchange of information on maternity-related subjects such as childbirth [20].

With regard to the study limitations, it should be noted that the health professional network was grouped together by professions (doctors, nurses, midwives), the TBA network anonymously and individually, and the relatives network according to relationship. However, given the study subject, it was necessary adopt this approach. Another of the limitations to bear in mind was the small sample size. The last limitation, the possibility of cognitive bias. Nevertheless, in this regard, people tend to forget weak ties, instead of strong ones (something that would favor our results).

Nevertheless, our SNA of a community and a past event has allowed us to retrieve, reflect on and highlight some essential and universal aspects of delivery care that remain the subject of debate today. Hence, we suggest that future research should conduct historical ethnography studies in conjunction with SNA. This would shed light on past social networks, yielding a better understanding of these and indicating key aspects to consider in the present. 

## 5. Conclusions

We have achieved our study objectives: (i) we have confirmed the transition from a home birth model of care to a scenario in which deliveries began to occur almost exclusively in a hospital setting; (ii) we have defined the social networks surrounding home births; (iii) we have analysed the influence of geography on the social networks surrounding home births.

Retrospective analyses of health and social care systems such as those described here reveal essential and universal aspects of delivery care that are still relevant today. These include: (a) the importance of health professionals in home delivery care; (b) the importance of the mother’s primary network; and (c) the decisive influence of the geographical location of the actors involved in delivery. All of these factors must be taken into consideration when developing strategies for maternal health: the characteristics of the geospatial context, the mother’s social support network and the social and community resources deployed.
